# Peptidoglycan Perception in Plants

**DOI:** 10.1371/journal.ppat.1005275

**Published:** 2015-12-17

**Authors:** Andrea A. Gust

**Affiliations:** Center for Plant Molecular Biology (ZMBP), Department of Plant Biochemistry, University of Tübingen, Tübingen, Germany; THE SAINSBURY LABORATORY, UNITED KINGDOM

## Introduction

Higher eukaryotes have evolved mechanisms to detect and discriminate between a wide range of commensal and infectious microorganisms. The first inducible line of defence common between plant and animal systems is microbe-associated molecular pattern (MAMP)-triggered immunity (MTI) [1,2]. MTI is based on the recognition of certain structurally highly conserved and, for the microbe, indispensable patterns that are usually not present in the attacked host organism. In plants, these MAMPs are detected at the cell surface by pattern recognition receptors (PRRs) [3,4]. Results from recent years indicate that PRRs are part of larger protein complexes, most of which associate only in the presence of the respective MAMP [5]. Subsequently, host defences are mobilised, eventually leading to a limitation of pathogen spreading and multiplication.

## Peptidoglycans Are Immunogenic in Plants

Peptidoglycan (PGN), or murein, is one of the most widespread carbohydrates in nature [6]. As a rigid component, it is present in almost all bacterial cell walls, except those of Archaea, and contributes to bacterial shape [6]. Structurally, PGNs are glycan polymers of alternating β (1,4)-linked N-acetylglucosamine (GlcNAc) and N-acetylmuramic acid (MurNAc) residues that are cross-linked by short peptide bridges, the exact composition of which depends on the bacterial species [6,7].

Due to its unique composition and its restricted occurrence in bacteria, plants and animals have evolved PGN perception systems to monitor the presence of bacteria [6,7]. Indeed, PGN has been established as a MAMP in model plants such as *Arabidopsis thaliana*, rice, and tobacco [8–12]. Notably, in contrast to animal systems in which minimal PGN motifs (such as muramyl dipeptide or tracheal cytotoxin) can trigger immunity-associated responses [6], plants rather recognize longer fragments of the PGN sugar backbone [10], although the exact fragment size required for immunogenic activity still remains to be determined. Typical immune responses stimulated by PGN treatment comprise an increase in cytoplasmic calcium concentrations, the accumulation of reactive oxygen species, the activation of mitogen-activated protein kinases, and transcriptional reprogramming [9,10]. Moreover, tomato plants pre-treated with PGN acquire an increased resistance to subsequent bacterial infection [13], an effect called priming, that is typically caused by MAMPs. Hence, PGN can be added to the list of microbial structures recognized in plants.

## PGN Is Perceived in Plants via LysM Proteins

**Fig 1:**
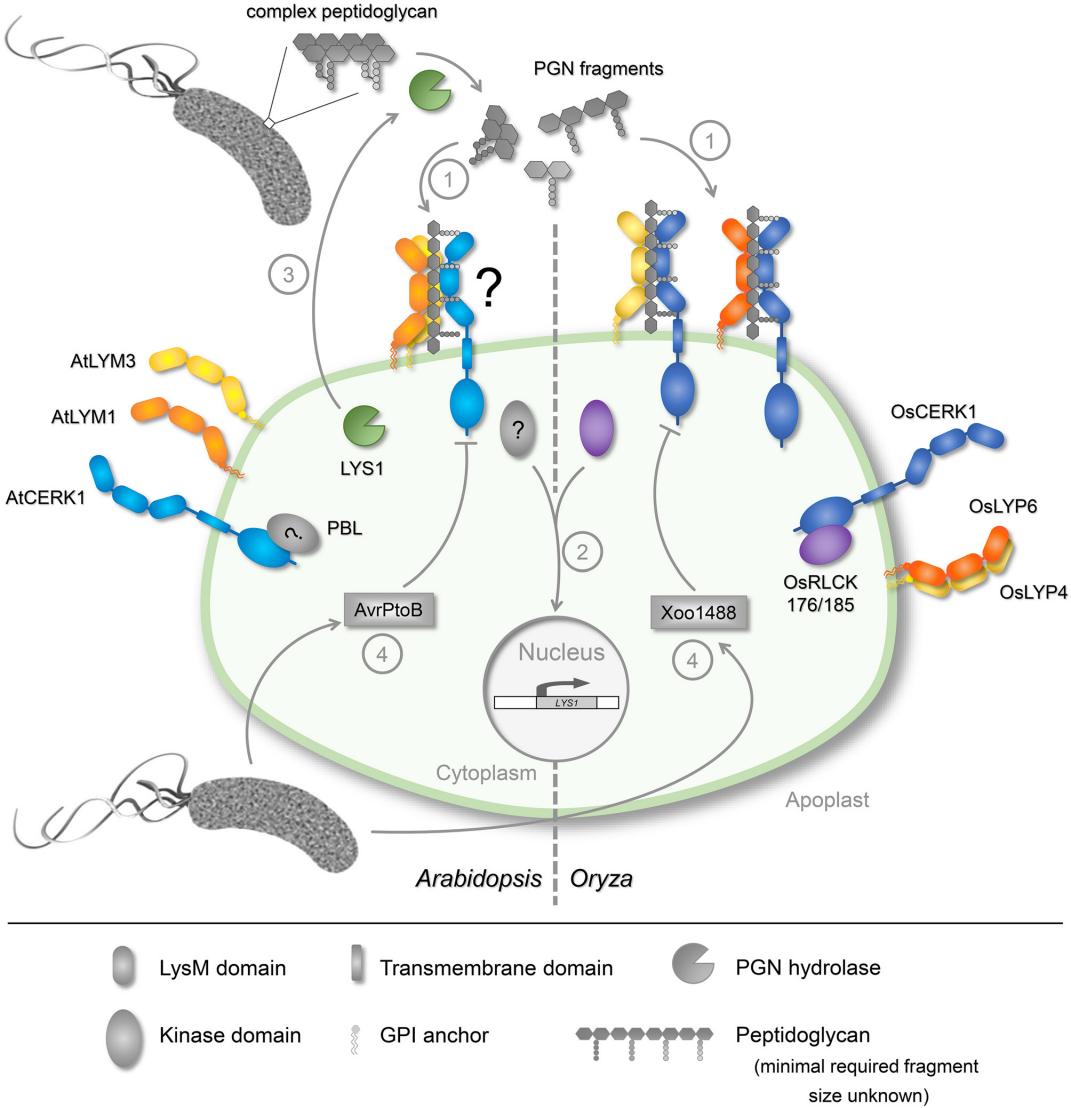
Overview of known components of PGN perception in plants. Upon entry of bacteria into the plant apoplast, their presence is most likely sensed either by PGN fragments spontaneously released into the apoplast or via unrelated bacterial MAMPs, such as flagellin or lipopolysaccharides. PGN fragments of yet unknown size bind to GPI-anchored LysM-proteins localized in the plasma membrane (AtLYM1/3 in Arabidopsis, OsLYP4/6 in rice). Upon PGN binding (1), OsLYP4/6 dissociate to form a complex with OsCERK1, which triggers the release of cytoplasmic OsRLCK176 (and possibly OsRLCK185) and subsequent downstream signalling (2). In Arabidopsis, AtLYM1, AtLYM3, and AtCERK1 mediate PGN perception (1); however, whether a PGN-dependent LysM protein complex is formed and whether receptor-like cytoplasmic kinases (RLCKs), such as avrPphB sensitive 1-like proteins (PBLs), are required is still unknown. Downstream signalling events lead to a transcriptional reprogramming of the cell, and defence proteins, such as the lysozyme-like activity LYS1, are produced. LYS1 is secreted into the plant apoplast to generate more immunogenic PGN fragments (3), which results in an amplification of PGN-triggered immune responses. In a successful infection, however, CERK1 is targeted by bacterial effectors such as AvrPtoB (in the *Arabidopsis*–*Pseudomonas syringae* interaction) and Xoo1488 (in the rice–*Xanthomonas oryzae* interaction) to suppress plant immunity (4).

In animal systems, several perception systems for PGN have been elucidated, including peptidoglycan recognition proteins (PGRPs, PGLYRPs), nucleotide-oligomerization domain protein 1 (NOD1) and NOD2, and, possibly, toll-like receptor 2 [6,7]. However, plants do not possess PGRP-like proteins, and plant cytoplasmic NOD-like proteins are involved in the detection of intracellular microbial effector molecules but not in MAMP recognition [14]. Rather, all plant receptors described, so far, to participate in the binding and/or perception of GlcNAc-containing carbohydrates (including PGN, chitin, or the chitin-related symbiosis factors) belong to the family of lysin-motif (LysM) proteins [15]. The LysM is an ancient motif that was initially identified in bacteriophages and bacterial proteins associated with PGN turnover but is now appreciated as a commonly used motif to mediate binding of GlcNAc-containing structures [16]. Plants like Arabidopsis contain various families of LysM proteins, such as LysM receptor kinases, membrane-anchored LysM proteins without an intracellular signalling domain, extracellular LysM proteins without a membrane anchor, and intracellular non-secretory LysM proteins [15], all of which potentially could function as PGN binding proteins. Indeed, the first plant PGN receptors to be identified were Arabidopsis LysM-domain protein (LYM) 1 and AtLYM3—two GPI-anchored, PGN-binding proteins located in the plasma membrane—and the LysM-receptor kinase chitin elicitor receptor kinase 1 (AtCERK1), which does not bind to PGN but is required for signal transduction (Fig 1) [17]. Importantly, AtLYM1 and AtLYM3 are specifically required for PGN perception and, unlike animal PGN receptors, they do not seem to discriminate between PGN from Gram-negative and Gram-positive bacteria [10,17]. The second plant species in which PGN receptors have been identified is rice, in which the LysM proteins OsLYP4 and OsLYP6, together with OsCERK1, are required for PGN and chitin recognition [12,18]. In the absence of PGN, OsLYP4 and OsLYP6 constitutively interact, although the function of this preformed complex is still unclear, and dissociate upon PGN treatment to join a complex with OsCERK1 (Fig 1) [18]. Whether only OsLYP4 or OsLYP6 (or possibly both) proteins are found in complex with OsCERK1 upon PGN binding is unknown. But, considering that AtLYM1 and AtLYM3 act in a cooperative manner [17], it can be assumed that both proteins are most likely part of the CERK1 complex at the same time (Fig 1). Also, the requirement of longer PGN fragments for immune-stimulation implicates a polymerization of several PRR molecules along the PGN molecule, as has been demonstrated for AtCERK1 and OsCEBiP, respectively, in chitin perception [5] and insect peptidoglycan recognition protein-SA in PGN recognition [19]. However, the formation of such tripartite AtLYM1/AtLYM3/AtCERK1 or OsLYP4/OsLYP6/OsCERK1 complexes is yet to be demonstrated.

Intriguingly, and with just a few exceptions (such as OsLYP4 and OsLYP6), all ligand-binding LysM proteins discriminate between specific GlcNAc-containing ligands, the basis of which is currently still unknown. A putative mechanism recently put forward and based on the co-crystallization of LysM proteins together with chitin fragments was the cooperative binding of multiple LysM domains to a glycan strand to confer carbohydrate specificity [20,21]. In this respect, it will be interesting to see whether other proteins that were recently implicated in chitin perception, such as LysM RLK1-interacting kinase 1 (LIK1) [22] or LysM-receptor kinase 4 (LYK4) and LYK5 [23,24], also serve a function in PGN detection.

Signalling cascades downstream of the identified PGN receptors are most likely activated via members of the RLCK family. In rice, OsRLCK185 and OsRLCK176 are required for PGN and chitin sensing and OsCERK1-bound OsRLCKs dissociate upon ligand perception to trigger immune responses (Fig 1) [18,25]. However, Arabidopsis RLCKs have so far only been implicated in chitin signalling [26,27], but evidence for their involvement in PGN sensing is still lacking.

## Plant Lysozyme-Like Activities Release Immunogenic PGN Fragments

Due to its rather complex structure, PGN most likely requires breakdown into smaller, more soluble fragments prior to binding to its plasma-membrane localized plant receptors. In living bacteria, PGN is very rigid; however, it needs to be sufficiently dynamic to allow bacterial growth and replication. During these phases of remodelling, PGN fragments are constantly shed into the bacterial environment, with the amount depending on recycling efficiency [7]. Released PGN fragments do not only give feedback to the bacteria themselves about the status of their cell wall, but have also been shown to serve as MAMPs in animals [6,7]. However, these rather small PGN fragments are unlikely to be effective as immunity triggers in plants [10]. Alternatively, host hydrolytic activities could directly target the bacterial cell wall to release PGN fragments with immunogenic properties [7,28,29]. Such enzymes include animal PGRPs with amidase activity, mammalian lysozymes, and plant lysozyme-like activities. Similar to lysozyme, which was shown to deliver PGN fragments to the cytoplasmic PGN receptor NOD2 [28], the Arabidopsis lysozyme-like activity LYS1 produces PGN-breakdown products with immunogenic activity, and *lys1* mutant plants are compromised in their resistance to bacterial infection (Fig 1) [29]. Thus, eukaryotic hosts most likely make concerted use of PGN hydrolytic activities and of PRRs in order to cope with bacterial infections.

## Phytopathogenic Bacteria Can Interfere with PGN Perception

Since PGN is such an important immunogen, bacteria have evolved multiple strategies to evade its recognition, most of which have been well described for animal pathogens. One of the first and most effective measures is to simply avoid the perception of PGN—for instance, by structural alterations of the perceived epitope(s). Indeed, some animal-pathogenic bacteria modify their PGN, sometimes even in the course of infection, to avoid clearance by the immune system [30]. Interestingly, muropeptides from phytopathogenic *Xanthomonas campestris* displayed higher immunogenic activity in plants than *Agrobacterium tumefaciens* muropeptides, and indeed, structural differences in the PGN of both bacteria were found [9].

Another strategy to avoid PGN perception is the suppression of the generation of immunogenic PGN fragments by PGN hydrolytic activities [28,29]. This can be achieved by structural alterations of the PGN; for instance, pathogenic staphylococci contain an additional O-acetylation of the muramic acid in the glycan backbone, rendering this PGN resistant to lysozyme [31]. Alternatively, some bacteria produce highly specific and potent lysozyme inhibitors [32]. These inhibitors can be anticipated to modulate the host immune response by interfering with the release of immunogenic PGN fragments during infection and thus contribute to host colonization by protecting bacteria against lysozyme challenge. However, inhibitors that are active against plant PGN-hydrolases such as Arabidopsis LYS1 [29] have so far not been described. Interestingly, some phytopathogenic fungi secrete effectors to shield their chitin shell against plant chitinases or to sequester released chitin fragments to prevent their binding to the chitin receptor CERK1 [33,34]. Future studies will determine whether some of the bacterial LysM proteins, most of which are secreted and bind to PGN [16], have such a function as PGN scavengers.

Last but not least, if a bacterial pathogen cannot avoid the generation of immunogenic PGN fragments or their binding to the PRR, bacterial effectors injected directly into the plant cytoplasm could actively suppress the host immune response. Importantly, both in Arabidopsis and in rice, CERK1 is a key player in PGN recognition [17,18], and several bacterial effectors have been shown to interfere with CERK1-mediated signalling [25,35]. For instance, Arabidopsis CERK1 protein stability is modulated by the *Pseudomonas syringae* effector AvrPtoB, an E3 ligase, by ubiquitinating CERK1 and thus facilitating its degradation [35]. In rice, the effector Xoo1488 from the bacterial pathogen *Xanthomonas oryzae* pv. *oryzae* was demonstrated to suppress PGN and chitin-induced defence responses [25], most likely by inhibiting the phosphorylation of OsRLCK185 by OsCERK1.

## Concluding Remarks

PGN has very unique characteristics and, hence, plants and animals have exploited this structure to monitor the presence of bacteria. Whereas good progress has been made during recent years concerning the identification of plant PGN receptors, there are still many open questions to be addressed in the future. For instance, are there structural features in phytopathogenic versus non-pathogenic bacterial PGN that determine immunogenic activity, and what is the minimal PGN motif-conferring activity in plants? How can LysM domains discriminate between GlcNAc-containing ligands? Are there bacterial inhibitors and effectors targeting plant PGN hydrolases or PGN sensors? Some gaps might be filled by learning from plant chitin recognition or from PGN perception in animal systems. However, fungal and bacterial pathogens follow distinct infection strategies, and often MAMP perception machineries only share limited similarity in animals and plants. For instance, animal PGN receptors do not possess LysM domains, but rather make use of PGRP domains (which cannot be found in plants) or leucine-rich repeat domains for PGN binding [7]—a motif that, in plants, was so far exclusively associated with the perception of proteinaceous ligands [15]. Thus, studies on PGN perception in plants will not only enhance our knowledge on plant glycan perception in general but will also help in drawing a more complete picture of the differences and similarities of MAMP perception systems in the two kingdoms.
